# Pivot Teeth

**Published:** 1851-04

**Authors:** J. Taft

**Affiliations:** Xenia


### PIVOT TEETH.

BY J. TAFT, D.D.S., XENIA.

The operation of inserting pivot teeth has been considered by many as one scarcely admissible, alleging that it is without a scientific basis; experience, however, shows us that this operation may frequently be performed with the happiest effect. To the ordinary method of inserting pivot teeth, there are many serious objections which cannot be overcome while that method is pursued; the tendency to rapid decay, and the consequent irritable condition of the contiguous parts, and the frequent production of abscess, the offensive odor, and vitiated condition of the mouth, are objections, as intimately connected with the common method as cause and effect can well be. There are other objections that might be named, but the above are the principal ones, and those most commonly referred to. The rapid decay of the roots of teeth bearing artificial crowns, is occasoned by their continued exposure to the action of the fluids of the mouth, and that in a vitiated condition; this exposure isn no way prevented by the common wood pivot.

The irritable condition of the contiguous parts is in almost all cases produced by *decaying* roots of teeth, those roots that remain sound, do not, with few exceptions produce irritation. Without irritation there is no abscess. The offensive odor is either a consequence of the decaying condition of the root or of an accumulation of vitiated foreign matter in the joint between the crown and the root. The offensive condition of the mouth may arise from the same cause. Any method by which these difficulties can be overcome would add much to the value of pivot teeth. This can be almost if not altogether accomplished by the use of the gold tube, and a gold substitute for the wood pivot. This operation though it has for the most part been described, is not yet practised to any extent. The gold tube or hollow wire can sometimes be obtained at the dental depots; but it is difficult to get it, as it is wanted in every case. The manner in which it is prepared is very simple: take a piece of common gold plate medium thickness, and about three and a half lines in width, and of any desirable length, and close it round a steel wire, just the size the pivot is designed to be; close it as perfectly, with the hammer, in the groove as possible, then anneal and draw it through the draw plate, with the steel wire still within it, until it is perfectly smooth and even. The tubes may now be sawed off at the proper length, and one end and the joint soldered: next smooth off the soldered parts, and then with the screw plate put a sharp fine screw thread upon it. The tube is now ready to be inserted; the root having been prepared and drilled out to the proper size, the tube having a piece of steel wire in it, is grasped with a pair of small pliers, with a slide to fasten them upon it; it is very carefully screwed into the cavity of the root, until it is to the bottom. Great care must be exercised in this part of the operation; after the tube is in, if the root was perfectly sound, with a small fine saw, the projecting part of the tube is cut off, and then with a very fine file dress it smooth with the root, and then burnish it and the end of the root until they are perfectly polished. I do not think it necessary to use the screw top in the root before inserting the tube, for it enters equally as easily without the top, and introducing the top would only cause a greater amount of irritation; it is very apparent that it would be almost impossible to make the thread of the tube follow that made by the top in the root; and if one should cross the other, the operation would not be so perfect; the tube will cut its way quite as ready as the screw top. It is oftentimes the case that the bone of the tooth, at the orifice of the cavity, even after the fang has been dressed off, is decomposed; when this is the case the decayed portion should be perfectly removed with the excavator—and after the tube is inserted and before it is cut off, the excavation should be perfectly filled with gold, and then all dressed and burnished together. A root prepared in this way is in much the same condition as a plugged tooth, except that a small portion of the tooth bone is exposed, but this, if well burnished, will resist decay for a long time. Here there is no cavity exposed, no lodgment for vitiated matter.

The root is now ready to receive the artificial crown which must be furnished with a pivot of gold; having the pivot wire of the right size, a portion of the hollow wire of which the tube is made, should be cut off equal in length to the depth of the hole in the artificial crown, into this place the first, and after applying ground borax, insert both into the crown, and then solder. The blowpipe must be used with great care to prevent cracking the tooth; for soldering, the tooth should be placed upon a piece of charcoal, and not in plaster. In directing the flame upon it, it should be thrown upon the body of the tooth and not upon the pivot, that the solder may flow down to the bottom of the cavity.

Pivots thus put in are as firm as if put in at the formation of the tooth. After dressing the pivot at the neck of the tooth where the solder may have flowed, it is ready to be inserted. Teeth thus inserted can be removed at pleasure, and may be kept as free from offensive accumulations, and odors, as artificial teeth under any other circumstances.

If this method of inserting pivot teeth was adopted by all good dentists, it would add very much to the character of this operation which has been at a low ebb.

The principal objection, by dentists, is the amount of time and labor required for its performance; this, however, is an objection only with those who have but seldom attempted it. It is in this as in other things, familiarity gives facility of execution.

---

### MASTICATION.

BY DR. E. W. POWER.

The special function which the teeth are designed to perform in the animal economy, is the prehension and the mastication of our food. We find them most skilfully adapted to this purpose by the peculiar shape of the different teeth, and their general arrangement in the jaws. The front teeth are bevelled down to a cutting edge, like that of an axe, for the purpose of grasping and dividing the food as it is presented to the mouth, while the molar or jaw teeth possess a broad surface divided into several prominences by transverse indentations, to fit them for breaking down the constitutent texture of the aliment and triturating it until it is properly prepared to be passed into the stomach, where it undergoes the main process of digestion.

For the due performance of the process all physiologists agree that the preparatory mastication of the food and its thorough mixture with the saliva are absolutely essential. It corresponds to the preliminary trituration to which the chemist would submit any solid matter that he might present it in the most advantageous form to a digestive menstruum. It reduces the food to that condition by which it is rendered more susceptible of being acted upon by the solvent powers of the gastric juice.

Mastication thus becomes the initial step in the important function of digestion, without the proper execution of which the ultimate conversion of the aliment into the various tissues

